# Self-reported hard physical work combined with heavy smoking or overweight may result in so-called Modic changes

**DOI:** 10.1186/1471-2474-9-5

**Published:** 2008-01-14

**Authors:** Charlotte Leboeuf-Yde, Per Kjær, Tom Bendix, Claus Manniche

**Affiliations:** 1 The Back Research Center, Part of Clinical Locomotion Science, University of Southern Denmark, Lindevej 5, DK-5750 Ringe, Denmark

## Abstract

**Background:**

Recently, the MRI finding of "Modic changes" has been identified as pathologic spinal condition that probably reflects a vertebral inflammatory process (VIP), which coincides with spinal pain in most. We hypothesized that heavy smoking in combination with macro- or repeated microtrauma could lead to VIP. The objectives were to investigate if combinations of self-reported heavy smoking, hard physical work, and overweight would be more strongly linked with VIP than with other spinal conditions, such as degenerated discs and non-specific low back pain (LBP).

**Methods:**

Secondary analysis was made of a data base pertaining to a population-based cross-sectional study. A population-generated cohort of 412 40-yr old Danes provided questionnaire information on smoking, weight, height, type of work, and LBP. MRI was used to determine the presence/absence of disc degeneration and of VIP. Associations were tested between three explanatory variables (type of work, smoking, and body mass index) and four outcome variables (LBP in the past year, more persistent LBP in the past year, disc degeneration, and VIP). Associations with these four outcome variables were studied for each single explanatory variable and for combinations of two at a time, and, finally, in a multivariable analysis including all three explanatory variables.

**Results:**

There were no significant associations between the single explanatory variables and the two pain variables or with disc degeneration. However, VIP was found in 15% of non-smokers vs. 26% of heavy smokers. Similarly, VIP was noted in 11% of those in sedentary jobs vs. 31% of those with hard physical work. Further, the prevalence of VIP in those, who neither smoked heavily nor had a hard physical job was 13%, 25% in those who either smoked heavily or had a hard physical job, and 41% in those who both smoked heavily and worked hard. The odds ratio was 4.9 (1.6–13.0) for those who were both heavy smokers and had a hard physical job as compared to those who were classified as "neither". Similar but weaker findings were noted for the combination of overweight and hard physical work but not for the combination of smoking and overweight.

**Conclusion:**

Hard physical work in combination with either heavy smoking or overweight is strongly associated with VIP. If this finding can be reproduced in other studies, it may have consequences in relation to both primary and secondary prevention of LBP, because blue collar workers, who are most likely to experience the consequences of LBP, also are those who are most likely to smoke.

## Background

### What is it that causes low back pain in the lower social classes?

Low back pain (LBP) is a common yet puzzling condition. Notably, its causes are not well established. Like so many other diseases, LBP is a problem particularly among people in the lower social classes [1]. But what, specifically, causes LBP in the lower social classes?

### Hard physical work?

Hard physical work is obviously suspected, as it could cause wear and tear on cartilage, tendons, discs, and muscles. However, many researchers seem to have become discouraged in relation to the search for the primary patho-anatomical causes of LBP [2,3] and opinion has veered more towards psycho-socio-economical issues particularly in relation to disability and the consequences of LBP [4].

### Smoking or overweight?

Other factors, also often determined by social class, such as being a smoker ("smoking") and having inappropriate eating habits, resulting in overweight ("overweight"), have been investigated in numerous studies over the past 30 years in relation to LBP. Smoking has been found to be positively associated with non-specific LBP in a large number of studies but this association – when present – is typically weak. Because strong evidence is lacking in relation to temporality and dose-response, causality cannot be assumed [5,6]. Furthermore, there is no convincing association between smoking and non-specific LBP in monozygotic twin pairs who are discordant on smoking [7].

There is also no obvious association with non-specific LBP in monozygotic twin pairs who are discordant on body weight [8]. As there is an absence of robust evidence of strength of association, temporality, and dose-response between weight and LBP [9], it could be concluded that evidence for a biological cause is lacking also for obesity. It is therefore possible that smoking and obesity are only proxy measures of some other underlying factors that are the true causes of LBP.

Nevertheless, several hypotheses in the literature address the rationale for a biological and causal link between both smoking and overweight and lumbar spine disease. For smoking, these are related to increased intra-abdominal pressure because of chronic cough [10], hormonal and/or other alterations [11], reduced blood flow to discs [12], diminished mineral content of bone resulting in painful microfractures of the trabeculae [13], and a fibrinolytic defect causing disc disease [14]. Other possible causes have also been suggested that are mainly non-biological [15-19]. It is certainly biologically plausible that also overweight could cause or contribute to LBP; both mechanical and systemic changes have been suggested [20].

Both smoking and overweight have previously been suspected of causing disc degeneration. We found, for example, studies on clinically diagnosed disc degeneration in the general population [14,20,21], in specific working populations [22-24], and in patient populations [25-31]. Similarly, the association between sciatic pain (which probably can be considered a proxy measure for disc degeneration) has been studied in the general population [13,32-35], specific working populations [10,17,36-39], and in patients [40]. The overall results of all these studies have been inconclusive. There are many possible explanations for this inconclusiveness, one of which is that the genetic background may be much more important than life-style [41-43]. It is also possible that there are some racial differences, which would result in varying results across continents, countries and subpopulations.

### MRI-identified vertebral marrow changes – a new diagnosis

A "new" MRI-defined entity is also worth considering in this context. De Roos et al [44] in 1987 and Modic et al in 1988 [45] were first to describe signal changes due to increased vascularity in the vertebral bone marrow extending from disrupted and fissured vertebral endplates. The disrupted endplates were confirmed by Toyone in 1994 [46].

Modic et al distinguished between two different types thought to be clinically relevant. The type 1 lesion reflects hypervascularity of the vertebral body and endplates, and the type 2 lesion consists of fatty replacements of the red bone marrow. These changes are believed to be signs of a vertebral inflammatory process (VIP). In this article, we use the same classification.

VIP can be a painful condition, both in patients and in the general population. Braithwaite reported that almost all patients diagnosed with discogenic pain reported pain on discography if they also had VIP but not so in those without VIP [47]. In a general population consisting of 40-year old Danes, 88% with VIP (type 1 or type 2) reported having had LBP in the previous year [48]. It has also been shown that persons with both disc degeneration and VIP were more likely to report having had LBP in the past week, month or year than those with disc degeneration but no VIP and those with neither disc degeneration nor VIP [49].

The prevalence of VIP (type 1 or type 2) was 22% in the Danish study [48]. In a somewhat older group of Finnish male workers the presence of VIP was considerably higher, consisting of a larger prevalence of type 2 lesions [50].

That VIP is indeed an inflammatory process has been shown by Ohtori in a histologic study of endplates in patients with and without VIP [51] and appears to have been further confirmed in another study. Butterman showed that patients with disc degeneration responded more favourably to epidural and intradiscal steroid injections if they had signs of VIP than if they had no signs of VIP [52]. For most, however, improvement was not lasting in nature. Indeed, a previous 3-year follow-up study of 60 unoperated patients with sciatica revealed that VIP in many cases is a long-lasting condition [53].

Why VIP should arise is not known, but the fact that it usually occurs in the lower lumbar spine [54,49] and together with disc degeneration [44,45] indicates that it could be caused by a chemical irritation from the degenerated disc [47] or that impaired biomechanics of the intervertebral segment is the cause [49].

Perhaps degenerated discs make the adjacent vertebrae vulnerable to trauma or repeated micro trauma, leading, in some cases, to micro fractures. Hansson and co-workers have shown in cadaver studies that both the endplate and the adjacent subchondral spongy bone of the vertebral body can fail under vigorous physiological loading conditions and that the location of these fatigue fractures corresponded to the location of earlier microcalluses in the vertebral body [55]. Further, the number of calluses increased with decreasing bone mineral content [56]. They also noted a correlation between smoking and the bone mineral content of the lumbar vertebrae in patients with chronic LBP [57]. Micro fractures may not be detectable on ordinary X-rays [58] and VIP is only visible on MRI, which explains why this has not been studied much in the past.

### Could smoking and hard physical work or smoking and overweight cause vertebral marrow changes -a biomechanical explanation?

We therefore propose a biomechanical explanation of why people in the lower social classes are more prone to LBP than others. This could be related to three common aspects of their life: type of work, smoking and dietary habits [1]. Heavy smoking, which results in reduced bone mineral content, could make vertebrae more prone to develop microfractures. Hard physical work may cause vertebral injuries in those with degenerated discs and a weakened bone structure, resulting in VIP. When an injury has occurred, it is also possible that the healing process is delayed in smokers. Overweight could also result in fatigue fractures of the vertebrae. Therefore a higher prevalence of VIP would be expected in people who either smoke heavily and work physically hard, or smoke heavily whilst being overweight.

We were unable to find any studies in which the effect of smoking in combination with hard physical work or obesity was specifically studied for VIP. However, in a large study of high quality, Eriksen et al reported that a job involving heavy lifting and much standing was a strong predictor of LBP- but only in smokers. The reported odds ratio was unusually high for this type of study, 5.5 (95% CI 1.9–15.8), even after adjusting for a number of relevant extraneous variables [59].

This important finding requires further scrutiny. We therefore decided to make use of a data set consisting of high quality MRI findings, including information on disc degeneration and VIP, as well as information on smoking, body mass index and type of work. The aim of the study was to determine if smokers' backs are more vulnerable to hard physical work and overweight, from this pathoanatomical point of view. Because disc degeneration is an age-related phenomenon and VIP appears mainly in people with degenerated discs, it was important that our study sample consisted of only 40-yr olds.

### Assumptions to be tested

• We assumed (as seen in most previous studies) that associations between either heavy smoking, overweight or hard physical work when tested one by one for a relationship with self-reported LBP would be weak or non-existent.

• Our second assumption was that these associations would be weak also when tested against disc degeneration but that they might be somewhat stronger for VIP.

• Most importantly, we assumed that stronger associations with VIP would emerge when combinations of variables were tested. In particular, in heavy smokers, who subject their back to repetitive compressive forces, such as physically hard work, or who are overweight.

• However, we assumed that we would not identify strong associations for these combinations with smoking in relation to LBP in general or to disc degeneration.

## Methods

### Data collection

The study makes use of already existing data. Study design, sampling method, data collection and MRI procedure have been extensively described elsewhere [48]. A summary is provided in Table 1.

**Table 1:** Brief description of the study design, sampling method, data collection, and MRI procedure.

*Study design*
Cross-sectional study
*Sampling method*
In all, 625 40-yr olds born in Denmark and living in the county of Funen were randomly selected from the population register (every ninth person), of which 412 accepted to participate

*Data collection*
Information on low back pain, type of work, height and weight were collected in a questionnaire and through MRI during the period of June 2000 till February 2001. The type of work was self-reported and divided into four main groups, of which one was described as "hard physical work". An experienced radiologist described all images according to a set protocol. The interpretation of MRI images was made independently of the present study subject.

*MRI procedure*
MRI was performed with an open low field 0.2 T MR unit (Magnetom Open Viva, Siemens AG, Erlangen, Germany). T1- and T2-weighted sagittal and T1-weighted sagittal sequences were used. 1) A localizer sequence of five images, 40/10/40 degrees (TR/TE/flip angle) consisting of two coronal and three sagittal images in orthogonal planes, 2) Sagittal T1 weighted spin echo, 621/26 (TR/TE), 144 × 256 matrix, 300 mm field of view, and 4 mm section thickness, 3) Sagittal T2 weighted turbo spin echo 4609/134 (Tr/effective TE), 210 × 256 matrix, 300 mm field of view, and 4 mm section thickness, and 4) Axial T2 weighted turbo spin echo 6415/134 (TR/effective TE), 180 × 256 matrix, 250 mm field of view, and 5 mm section thickness in the plane of the five lower discs.

*Ethics*
The local ethics committee and the Danish Data Protection Agency approved of the study. All participants received written information and signed an informed consent.

### Variables of interest

#### Outcome variables

Variables relevant for the present study were: LBP in the past year (LBPyear), LBP for altogether >30 days in the past year (LBPlong), disc degeneration (DD), and VIP (Table 2).

**Table 2:** A description of how the variables of interest were defined and grouped.

1. Outcome variables	1. LBP some time in the past year, "LBPyear" (yes/no)
	2. LBP for altogether at least 30 days in the past year, "LBPlong" (yes/no)
	3. Disc degeneration, "DD" (yes/no)
	4. Disc degeneration with VIP, "VIP" (yes/no)
2a. Single explanatory variables	1. "Smoking", divided into 0, 1–19 cigarettes/day, and ≥20 cigarettes/day.
	2. "Body mass index" (BMI) divided into BMI <20 ("underweight"), BMI 20–24 ("normal weight"), BMI 25–29 ("overweight"), and BMI >29 ("heavy overweight").
	3. "Work", divided into mainly sitting, sitting/walking, light physical work, and hard physical work

2b. combinations of the explanatory variables	Based on the results obtained with the single explanatory variables, the following variables were created:
	1. "Heavy smoking" (yes/no); i.e. smoking divided into ≥20 cigarettes a day vs. 0–19 cigarettes/day.
	2. "Hard physical work" (yes/no); i.e. hard physical work vs. the other three types.
	3. "Overweight" (yes/no); i.e. overweight and heavy overweight vs. normal weight and underweight.

The LBP variables were obtained from a questionnaire. The definition of disc degeneration as seen on MRI was: Reduced disc height (grade 2 or 3) or disc signal grade 3 [60,61]. VIP was defined as an MRI-finding in the vertebral body extending from the endplates and regardless size, detected as a high signal on T2-weighted and low signal on T1-weighted images (type 1 lesion) or as a high signal on both T1- and T2-weighted images (type 2 lesion).

#### Explanatory variables

Questionnaire data on smoking, body mass index (BMI) and work were used as explanatory variables and analysed both singly and in combinations of two. Cut points for the combination variables were selected after preliminary analyses of the single variables vs. the outcome variables (as reported in the Result section). (For detailed definitions, see Table 2).

Each of these combination variables was then divided into three subgroups: *"neither *the one *nor *the other", *"either *the one *or *the other", and *"both *the one *and *the other".

For example, the new combination variable "heavy smoking and hard physical work" was subdivided into "neither heavy smoking nor hard physical work", "either heavy smoking or hard physical work", and "both heavy smoking and hard physical work".

### Data analysis

Associations were studied for *each single explanatory variable *in relation to each outcome variable (bivariate analysis). Significant differences between estimates were defined as those having non-overlapping 95% confidence intervals between the lowest and highest estimates (reported as percentages).

Thereafter, the pattern of differences was studied for the *combinations of the explanatory variables *("heavy smoking/hard physical work", "heavy smoking/overweight", and "overweight/hard physical work") in relation to subgroups of "neither", "either...or", and "both...and". (Subjects, who were heavy smokers, were overweight *and *reported hard physical work were too few to allow an analysis of all three explanatory variables at the same time.) Associations were reported as odds ratios (OR) with 95% confidence intervals.

Finally, a *multi variable logistic regression *was performed for each of the outcome variables, with the explanatory variables classified as heavy smoking (yes/no), work (4 categories), BMI (4 categories), also including sex. Significance was set at p < 0.05 and results reported as ORs with their 95% confidence intervals. This was done for all and independently for men and women.

### Validity

None of the explanatory variables had been submitted to any type of validation process and a certain degree of uncertainty must be expected as to their exactness in all individuals. For example, it is possible that overweight people overestimate the amount of physical work performed because they fatigue easily. It may also be difficult to fit ones work precisely into one of four categories, and even so, people may change jobs and tasks also vary over time. Nevertheless, the self-reported 4-category grouping has been used previously in a large Danish general population has previously produced logical answers [62] and appears to be a suitable means for a preliminary investigation, short of a very comprehensive work place description.

It may also be difficult to classify some people into specific smoke-categories. Some people are sometimes-smokers, others are ex-smokers and some even "recurrent ex-smokers", i.e. ex-smokers who sometimes revert to their old habit. Previous reviews have failed to show a big difference in LBP between current smokers and ex-smokers, nor did they reveal an obvious dose-response [5,6], and because we were mainly interested in those who smoked a lot, our main cut-point was between less than one packet a day and at least one packet. This is most likely a valid measure of current smoking habits but does not take into account the accumulated lifetime smoking habit. However, it is possible that the duration of the habit was similar for most smokers, as smoking usually starts at an early age and all participants in this study were aged 40.

The BMI measure may also have been incorrect for some, as it was based on self-reported weight and height. Most likely, this could result in an under reporting of weight and an over reporting of height, the consequence of which would be an underestimate of the associations between BMI and any other variable.

The long-term recall ability of the occasional experience of LBP is probably not good but people are more likely to remember long-lasting pain. The LBPlong variable is therefore most likely more valid than the LBPyear variable. However, their validity is not of utmost importance, as our study aimed at reproducing findings obtained in other studies that also used rather vague LBP variables. Nevertheless, a check for logical errors revealed a high proportion of consistent answers for those LBP questions that it was possible to check [63].

Our most important outcome variables, DD and VIP, however, were of excellent quality. They had both been subjected to intra- and inter-examiner tests, and found to be satisfactory [63]. For the disc parameters: K_w _was 0.56–0.87 and for VIP the percentage of agreement was 98% (intra-examiner reliability) and the K_w _was 0.6 (inter-examiner reliability). In addition, the interpretation of the MRI data was done independently of information relating to the hypotheses of the study.

Data collection was supervised by a radiographer and one of the authors (PK). Participants filled out their questionnaire independently of people with an interest in the study, thus reducing the risk of obsequiousness bias. The data base was checked for logical and factual errors. Data analysis was performed by the person (PK) who had collected the data and created the data base. In all, we were therefore confident that our data were satisfactory.

## Results

### Descriptive data

The study sample consisted of 412 persons, all aged 40, 52% of whom were women. The spread of data for the variables of interest is shown in Table 3.

**Table 3:** Description of the study sample consisting of 412 40-yr old Danes from the general population

*Sex*	
Men	199
Women	213
*LBP some time past yr (LBPyear)*	
Yes	284
No	128

*LBP altogether for at least 30 days past yr (LBPlong)*	
Yes	102
No	310

*Disc degeneration (DD)*	
Yes	214
No	198

*Disc degeneration with signs of marrow changes consistent with a vertebral inflammatory process, type 1 or type 2 lesion (VIP)*	
Yes	73
No	339

*Smoking*	
0 cigarettes/wk	169
1–19 cigarettes/wk	172
≥20 cigarettes/wk	70
missing data	1

*BMI*	
underweight	20
normal weight	214
overweight	115
heavy overweight	60
missing data	3

*Work*	
Mainly sitting	89
Sitting/walking	144
Light physical	92
Hard physical	84
Missing data	3

### Is smoking, work, or obesity on their own associated with the outcome variables?

#### Smoking on its own

All the estimated prevalence rates of LBPyear, LBPlong, DD, and VIP were larger for those who smoked ≥20 cigarettes/day as compared to the non-smokers. However, all confidence intervals were overlapping.

#### BMI on its own

The lowest estimates for each outcome variable was always noted for those who were underweight. The highest estimates were found for those who were overweight, whereas the estimates were lower in all cases for those who were heavily overweight. Again, these differences were non-significant.

#### Work on its own

Sitting at work was always associated with the lowest estimate regardless the outcome variable. Hard physical work was associated with the highest frequency estimate in three of the four variables. For VIP this gap spanned between 11% and 31%, with 95% confidence intervals that did not overlap.

#### Summary of findings

A low BMI and sitting at work, when viewed independently, appeared to be "protective" in relation to all outcome variables. Hard physical work, heavy smoking, and overweight, as single variables, were usually associated with the highest estimate for all four outcome variables. However, the only significant difference was that between sitting at work and hard physical work for VIP. There were no significant changes when the analyses were made independently for men and women (data not shown). For an overview of the results, see Table 4.

**Table 4:** Frequency table showing the associations between the explanatory variables and the four outcome variables for 412 40-yr old Danes (percent and 95% confidence intervals). Estimates with non-overlapping confidence intervals were considered to be significantly different from each other.

**Independent variables**	**LBPyear**	**LBPlong**	**DD**	**VIP**
*Smoking*				
None	64 (56–71)	27 (20–33)	51 (44–59)	15 (10–21)
1–19 cig/day	72 (65–79)	20 (14–26)	50 (42–57)	16 (11–22)
≥20 cig/day	73 (62–83)	33 (22–44)	57 (45–69)	26 (15–36)

*BMI*				
Underweight	50 (26–74)	15 (-2 -32)	40 (16–63)	10 (-4-24)
Normal weight	69 (62–75)	25 (19–31)	49 (42–55)	18 (13–23)
Overweight	70 (62–79)	28 (19–36)	59(50–68)	20 (13–27)
Heavy overweight	72 (60–83)	22 (11–32)	55 (42–68)	17 (7–26)

*Work*				
Sitting	67 (57–77)	20 (12–29)	43 (34–55)	11 (4–18)*
**S**itting/walking	67 (59–74)	22 (15–29)	58 (49–66)	19 (13–26)
Light physical	68 (59–78)	30 (21–40)	46 (35–56)	10 (3–16)
Hard physical	75 (54–84)	26 (17–36)	58 (48–69)	31 (21–41)*

*The non-overlapping confidence intervals between the "best" and the "worst" categories are written in bold.

LBPyear = LBP some time in the past year.

LBPlong = LBP for altogether at least 30 days in the past year.

DD = disc degeneration as defined in the methods section.

VIP = vertebral inflammatory process as defined in the methods section

### Are heavy smoking, overweight, and hard physical work in combinations of two associated with the outcome variables?

#### Heavy smoking and overweight

There was, for all outcome variables, a non-significant pattern of a positive gradient going from "neither" to "both".

#### Heavy smoking and hard physical work

Again, for all four outcome variables, there was a consistent dose-response pattern. In accordance with our assumptions, the estimates for VIP differed most, with a positive gradient going from 13% for those who were neither heavy smokers nor had a hard physical job, through 25% for those who were either heavy smokers or had a hard physical job, to 41% for those who both were heavy smokers and had a hard physical job (OR between the lowest and the highest estimates: 4.6; 95% CI: 1.6–13.0).

#### Hard physical work and overweight

Also for this combination there were positive gradients going from having neither factor to both factors. The strongest association was noted when comparing people who had neither factor with people who had both risk factors for VIP (OR 2.9; 1.4–6.3).

#### Summary of findings

Table 5 shows a similar and consistent pattern across the analyses but the results were sufficiently strong to be statistically significant only when hard physical work is involved and only for the more specific (pathological) variables, DD and VIP. Hard physical work in combination with heavy smoking had a particularly strong link with VIP. The combination of hard physical work and overweight was significantly linked with DD and with VIP, whereas no significant findings were noted for the combination of heavy smoking and overweight.

**Table 5:** The associations between combinations of explanatory variables and the four outcome variables (odds ratios and 95% confidence intervals). (N = 408)

**Combination of Independent variables**	**LBPyear**	**LBPlong**	**DD**	**VIP**
Heavy smoking and hard physical work				
*neither (n = 274)*	1	1	1	1
*either (n = 117)*	1.3 (0.8–2.1)	1.2 (0.7–2.0)	1.4 (0.9–2.2)	2.2 (1.3–3.8)*
*both (n = 17)*	1.6 (0.5–5.1)	1.8 (0.6–5.1)	1.5 (0.5–4.0)	4.6 (1.6–13.0)*

Overweight and hard physical work				
*neither (n = 193)*	1	1	1	1
*either (n = 171)*	1.3 (0.8–2.0)	0.9 (0.6–1.5)	1.6 (1.1–2.4)*	1.3 (0.7–2.3)
*both (n = 42)*	1.5 (0.7–3.1)	1.2 (0.6–2.6)	1.7 (0.9–3.4)	**2.9 (1.4–6.3)**

Overweight and heavy smoking				
*neither (n = 201)*	1	1	1	1
*either (n = 171)*	1.3 (0.9–2.1)	1.2 (0.8–2.0)	1.5 (1.0–2.2	1.4 (0.8–2.4)
*both (n = 36)*	1.2 (0.5–2.6)	1.5 (0.7–3.3)	1.6 (0.8–3.3)	1.6 (0.8–3.9)

LBPyear = LBP some time in the past year.

LBPlong = LBP for altogether at least 30 days in the past year.

DD = disc degeneration as defined in the methods section.

VIP = vertebral inflammatory process as defined in the methods section.

* Estimates that are statistically significantly different from "1" have been written in bold.

### Heavy smoking, hard physical work and overweight – the multivariable model (all participants)

In relation to VIP, the multivariable analysis confirmed that hard physical work is the "culprit", with an OR of 3.3 (1.4–7.6; p = 0.004). Heavy smoking had an OR of 1.9 (1.0–3.6; p = 0.051). Also BMI (regardless of category) and sex failed to reach significance.

## Discussion

### What did we find?

Largely, all our assumptions were met. In this general population of 412 40-yr old Danes, the pattern of findings in relation to LBP resembled that often found in the literature, namely positive but weak associations with smoking, BMI, and type of work as single variables.

The same was the case for DD. None of the three single explanatory variables on their own displayed a strong association with DD on its own. Also this finding was expected, as it has been shown that, at least, smoking and type of work – can explain only a small part of lumbar DD [64]. It has also been shown that the clinical findings of people with merely DD more closely resemble people with non-degenerated discs than people with both DD and VIP [49].

As anticipated, the picture differed for persons with VIP; almost three times as many of those with hard physical work had VIP compared with those with a sedentary job.

In addition, when the three sets of combinations of variables were studied in relation to VIP, it was revealed that those with hard physical work who were heavy smokers had an OR of 4.6 as compared to those who neither worked physically hard nor were heavy smokers. Furthermore, for people who both were in a heavy physical job and were overweight, the OR was 2.9, when compared with those who neither worked hard nor were overweight. In contrast, the combination of heavy smoking and overweight resulted in a non-significant estimate of 1.6, indicating that the hard work variable is the crux of the matter and not heavy smoking; a finding later confirmed in the multi variable analysis.

In summary, our significant positive findings related mainly to the outcome variable of VIP. The similar but much weaker and non-significant findings revealed also for the self-reported LBP variables can probably be explained by the fact that the association becomes "diluted" when including also subgroups of people with LBP from other causes than VIP.

### Strengths and weaknesses of the study

This study has several strengths. For example, the study subjects were obtained from the general population and they were all of the same age. The MRI was taken for research purposes, resulting in excellent images; the intra- and inter-examiner reliability has been previously tested and found to be satisfactory [63]. A more detailed discussion of the quality of data was included in the Methods section but in sum, any weaknesses in validity are likely to result in weaker rather than stronger associations. Contrary to most aetiological studies, explanatory and outcome variables were specifically selected in order to test specific assumptions and the data analysis was performed specifically with consideration for these hypotheses. A weakness of the study, however, is that the data set has already been used for other purposes, and the results therefore need to be tested in other studies. It is also possible that our BMI variable could have produced different results, if we had access to anthropometric data from early childhood, in order to study the long-term effect of overweight, as in the study by Liunke et al [20]. Their long-term weight data resulted in a much stronger association with MRI-defined disc degeneration than their prevalent weight data.

We did not take into account educational and other social factors that doubtlessly also have an influence on people's lifestyle, in addition to their self-reported type of work, BMI and smoking habits. This would also need to be taken into consideration, in future studies in this area.

### The biomechanical hypothesis

Although the direction of this link was not tested, it is likely that heavy smoking and hard physical work in combination are true risk factors for VIP because there is at least one biologically plausible explanation, the association is relatively strong, and there is a "dose-response". It is also likely that the explanatory variables preceded the outcome variable. In other words, although our data are not prospective in nature, these findings support the hypothesis of a biomechanical cause of VIP [65].

Our findings are in agreement with the biomechanical hypothesis also because the lowest prevalence estimates of VIP (10% and 11%) were found in those who were underweight, and in those who described their job as mainly sedentary. Generally, the prevalence of VIP was higher with increasing loads, in relation to body weight or type of work, and almost one-third of those who reported their job to be physically hard had VIP.

Further, overweight on its own was not as strongly linked with VIP as heavy physical work. This may also be an argument for the biomechanical hypothesis. Excessive body weight is a progressive condition, which allows the body to adapt and for bones to strengthen, whereas the compressive and shearing forces and loads experienced at work are external in origin and often beyond one's own control, and therefore potentially more detrimental.

The observation that hard physical work in combination with overweight also resulted in VIP indicates that heavy smoking is not a prerequisite, but that hard physical work is the main factor. In such a case, heavy smoking could be an additional aggravating factor, which makes recovery slow or impossible.

### Perspectives

From a public health perspective, it would be necessary to consider the consequences of present work/smoke practices, particularly among groups with heavy physical work. In addition, it would be important to study the time needed for restitution of discs and vertebrae following a day of heavy physical activities.

### Additional research considerations

Although it is likely that at least one missing link has been found that can explain the many but mainly weak associations between smoking/obesity and LBP in the literature, VIP may not be the only explanation. Consequently, it would be relevant to perform other hypothesis-generated studies of, for example, nicotine-induced vasoconstriction and its effect on muscles [66], smoke-induced altered nociception [67], and the genetic aspects of disc degeneration, bone density and their sequelae, taking into account also the possibility for ethnic differences.

## Conclusion

Over the past decades a large number of studies have been conducted, in which the association between smoking/overweight and LBP have been tested. However, these associations have typically not been very strong resulting in the conclusion that no causal link is apparent or, at least, that such a link is not clinically relevant.

The results of this study indicate that it is likely that the combination of hard physical work and heavy smoking, and to some degree also hard physical work and overweight, can result in a specific spinal pathology, VIP. However, these combinations did not result in significant associations with any of the pain variables.

As VIP is painful in many but present only in some people with LBP, the subpopulation with painful VIP would become submerged in the larger group of people with (other types of) non-specific LBP. This may explain the numerous weak to moderate associations between smoking/overweight and LBP, reported over the past thirty years.

If these findings can be reproduced in other studies, they could have considerable public health consequences.

## Competing interests

The author(s) declare that they have no competing interests.

## Authors' contributions

TB secured most of the funding for the MRI-project, was responsible for the main project and contributed to the manuscript. PK collected the data, analyzed the data in the present project, and contributed to the manuscript. CL-Y was involved with the main project, formulated the present research questions, interpreted the data, and wrote the manuscript for the present project. CM secured some of the funding, was involved with the main project, and contributed to the manuscript. All authors read and approved the final manuscript.

## Pre-publication history

The pre-publication history for this paper can be accessed here:


